# Effects of host plant on the development and reproduction of *Agrotis ipsilon* (Lepidoptera: Noctuidae) on horticultural crops

**DOI:** 10.1016/j.heliyon.2023.e17836

**Published:** 2023-06-29

**Authors:** Young Su Lee, Hee-A Lee, Gil-Hah Kim, Sunghoon Baek

**Affiliations:** a Department of Environmental Agriculture Research, Gyeonggi-do Agricultural Research and Extension Services, Hwaseong, 28333, Republic of Korea; b Department of Plant Medicine, Chungbuk National University, Cheongju, 28644, Republic of Korea; c Department of Agriculture and Fisheries Convergence, Korea National University of Agriculture and Fisheries, Jeonju, 54874, Republic of Korea

**Keywords:** *Agrotis ipsilon*, Host effect, Host fitness, Horticultural crop

## Abstract

One of cosmopolitan pest, *Agrotis ipsilon* (Lepidoptera: Noctuidae), causes serious economic damages in horticultural crops. This pest is difficult to manage and causes irreversible damage because its larvae stay in the ground at day and cut the plant stems at night. Thus, this study compared the host fitness of *A. ipsilon* among nine major horticultural crops in Korea. Among the nine crops, the population of *A. ipsilon* failed to complete its development in spinach, cucumber, melon, and kidney bean. The host effects on development and reproduction of *A. ipsilon* were further investigated in the remained five crops (i.e., napa cabbage, soybean, perilla, corn, and pepper). Host plants significantly (*P* < 0.05) affected the development-related factors (i.e., developmental time, survivorship, and weight) of *A. ipsilon* eggs, larvae, and pupae. They also affected the adult reproduction-related factors including preoviposition period, oviposition period and number, and longevity except for the prepupa stage. A positive relationship was found between biological factors (i.e., development- and reproduction-related factors). Among the nine crops in this study, napa cabbage showed the highest suitability for the *A. ipsilon* populations. These findings in this study would be helpful to understand the ecology and develop the management tactics of *A. ipsilon* in horticultural crops*.*

## 1 Introduction

One of notorious pest, *Agrotis ipsilon* (Lepidoptera: Noctuidae), is a worldwide distributed polyphagous insect [1,2]. The first and second instars mainly feed leaves of plants, but the damage is trivial in economic aspects [3]. From the third instar stage, its larvae stay in the ground at day and can cut the stems of young plants at night even though they still mainly feed leaves [3]. Thus, it is known as the “black cutworm”. Older instars (i.e., more than fifth instar stage) cause serious economic damage [4] in more than 100 plants including corn, wheat, cotton, bean, and grass [5]. The presence of the older larvae of *A. ipsilon* occur during the nursery stage of plants can lead to nearly 80% damage [6].

Even though *A. ipsilon* can feed more than 100 host plants worldwide [5], the economic damage is frequently reported in only a few crop plants such as napa cabbage, soybean, perilla, corn, pepper, spinach, cucumber, melon, and kidney bean in Korea. The reason is that the quality of host plants could affect the developmental factors (e.g., developmental rate, survivorship, weight, and so on), reproductive factors (e.g., pre-ovipositional period, ovipositional duration and number, and so on), and/or behaviors (e.g., attractance or repellence to the host, ovipositional location within the host, and so on) of its herbivores [7]. The effects of host plant on the populations of *A. ipsilon* were already reported in a few studies [[8], [9], [10], [11], [12]]. The four studies [[8], [9], [10], [11]] focused on the effects of host plants on *A. ipsilon* larvae without further study related with its reproduction. Even though the study of Muştu et al. [12] covered the reproduction of *A. ipsilon*, the host effects on *A. ipsilon* weight and survivorship were ignored. Until now, there was no study of host effects for *A. ipsilon* to cover all life stages and major biological factors.

This study focused on horticulture crops because the damage of *A. ipsilon* mainly occurred in these crops in Korea and previous studies [[8], [9], [10], [11], [12]] also indicated that *A. ipsilon* infestation of horticulture crops induced serious economic damage compared with other crops and weeds in other countries. Thus, this study investigated whether *A. ipsilon* completed its development from eggs to adults on nine major horticultural crops in Korea. The biological characteristics of each developmental stage (i.e., egg, larva, pupa, and adult) were investigated based on the host plants on which *A. ipsilon* successfully completed its development. Because the adults of *A. ipsilon* fly long distances [12], the relationships of biological characteristics between immature and adult stages were compared in this study. Based on the information provided in the present study, a potential management strategy for *A. ipsilon* was suggested.

## 2 Materials & methods

### 2.1 Experimental insects

This study used laboratory populations of *A. ipsilon*. These populations were derived from colonies collected across multiple peanut fields (N 36.55, E 126.33) in Dangjin-Si, Chungcheonnam-Do, Korea during May of 2018. Hundreds of larvae were reared on young napa cabbage (*Brassica rapa* subsp. *pekinensis*) leaves in plastic containers (21 × 27 × 12.5 cm) with artificially made ventilation meshes on the lid to separate *A. ipsilon* from other moths. The *A. ipsilon* populations were identified morphologically and continued until all collected larvae became adults. Morphologically ambiguous individuals were not selected. The identified and just hatched adults transferred to an acryl cage (30 × 30 × 30 cm, custom-made) with young napa cabbages to collect *A. ipsilon* eggs. To activate its oviposition, a mixture of honey and pine powder (1:1) diluted with a 10% sugar solution was provided. Oviposited eggs were collected every day and randomly transferred to one of nine plastic containers (21 × 27 × 12.5 cm) with ventilation meshes on the lid. Each plastic container was supplied with leaves from a single crop until third instar stage. The leaves that served as the food source for young larvae were obtained from nine major horticultural crops in Korea: napa cabbage, soybean (*Glycine* max subsp. *soja*), perilla (*Perilla frutescens* var. *frutescens*), corn (*Zea mays* var. *rugosa*), pepper (*Capsicum annuum* var. *annuum*), spinach (*Spinacia oleracea*), cucumber (*Cucumis sativus* var. sativus), melon (*Cucumis melo* var. *makuwa Makino*), and red kidney bean (*Phaseolus vulgaris*). Starting from the third instar stage, each larva was individually reared within an insect breeding dish (10 cm diameter × 4 cm height, SPL; Pocheon-Si, Korea). Fresh young leaves were provided daily to larvae individually. Among the nine host plants, adults successfully developed in five plants, napa cabbage, soybean, perilla, corn, and pepper. Within these five host plants, each population was maintained for more than five generations before experiment to ensure adaptation to host plant. All insect rearing and experiments described in this manuscript were conducted in an insectarium at 25 (±1) °C with a photoperiod of 16:8 (L:D) h and relative humidity (RH) of 65–75%. The host plants in this experiment were grown under similar environmental conditions in a separated plant rearing room.

### 2.2 Effects of host on egg development

The 30 pairs of *A. ipsilon* adults less than two days old in each host population were collected in an acryl cage (30 × 30 × 30 cm) with its own young plant hosts (napa cabbage, soybean, perilla, corn, and pepper). During the establishment of each host population, a mixture of honey and pine powder (1:1) diluted with a 10% sugar solution was supplied. In each host cage, the oviposited eggs within 24 h were collected into an insect breeding dish (10 cm diameter × 4 cm height). Newly hatched larvae were recorded, and transferred into insect breeding dish (10 cm diameter × 4 cm height) individually. The eggs, which were not hatched for more than three weeks, were considered as dead eggs.

### 2.3 Effects of host on larvae and pupae

In each host plant, the first 100 larvae emerging from the egg developmental experiment were observed. Individual breeding dishes were checked daily to provide young plant leaves and record biological changes of *A. ipsilon* larvae. When the larva in each breeding dish became prepupa, the food supply was stopped, and a moistened dental wick (Deahan Medical; Chungju-si, Korea) was provided to maintain suitable relative humidity within the breeding dishes. Further, the sex of each pupa was also observed and recorded. The pupa weight was separately measured for 50 males and 50 females emerging from the rearing cage of each population to ensure adequate numbers.

### 2.4 Effects of host on adults

A single pair of just hatched (less than a day old) adult male and female from the previous experiment was moved into the round acryl cage (9 cm diameter × 20 cm height, custom-made) with its own young host plant, except for the bean population. In bean, only seven adults successfully developed from the 100 larvae during the larval development. Thus, the newly hatched adults from the rearing cage were used for the bean population. The wall of the round acryl cage was covered with a meshed (1 × 1 mm) cotton gauze (15 × 15 cm) (Deahan Medical; Chungju-si, Korea) to collect *A. ipsilon* eggs. The mixture of honey and pine powder (1:1) was also provided by diluting it with 10% sugar solution as the food source. The meshed cotton gauze, host plant, and food were replaced daily and daily oviposition number and biological changes of adults were monitored.

### 2.5 Statistical analysis

Because the populations of *A. ipsilon* in this study were maintained during multiple generations in each host plant (napa cabbage, maize, pepper, bean, and perilla), its biological fitness (i.e., developmental rate, survivorship, pupa weight, oviposition period, and survival period) of each host was expected to be different according to reared hosts. Thus, the planned contrasts were applied in statistical analysis, not the post-hoc test. Except for survivorship, the *t-*test at 95% confidential level was used to determine host effects and sexual differences [13]. In the absence of host effects, ANOVA [13] was double-checked and described. The survivorship of each developmental stage was compared with chi-square test [13]. The relationships of host effects between biological factors in development and reproduction of *A. ipsilon* were analyzed with the linear regression [13].

## 3 Results

### 3.1 Effects of host on egg development

Despite less than a day difference to complete egg development of *A. ipsilon* depending on the host plant, the eggs of the population reared on perilla needed statistically (*P* < 0.05) more time than the other host species in this experiment (Table 1). By contrast, the eggs of napa cabbage populations required the least time to hatch among the five hosts (Table 1). However, the survivorship of eggs was significantly (*P* < 0.05) higher on napa cabbage, corn, and perilla than on pepper and bean (Table 2). The survivorship of *A. ipsilon* eggs was the least on the beans in this study (Table 2).

**Table 1:** Developmental time (days ± SE) of *A. ipsilon* immatures according to host.

Host	Egg		Larva		Prepupa		Pupa		Egg to adult	
	N^2^	D^3^	N	D	N	D	N	D	N	D
Napa cabbage	744	4.0 ± 0.01d^1^	95	20.8 ± 0.41c	92	2.6 ± 0.06a	85	14.5 ± 0.31ab	85	41.6 ± 0.47b
Corn	923	4.1 ± 0.01b	86	26.5 ± 0.55ab	83	2.4 ± 0.09a	61	13.6 ± 0.26c	61	46.2 ± 0.53ab
Pepper	572	4.2 ± 0.02bc	59	27.4 ± 0.30ab	58	2.5 ± 0.10a	54	15.1 ± 0.32a	54	49.0 ± 0.71a
Bean	164	4.0 ± 0.01cd	41	31.1 ± 0.71a	35	2.7 ± 0.12a	7	15.7 ± 0.52ab	7	53.1 ± 1.01ab
Perilla	365	4.5 ± 0.03a	64	25.2 ± 0.43b	60	2.9 ± 0.11a	46	15.5 ± 0.36a	46	48.5 ± 0.74a
Difference^4^	0.5		10.3		0.5		2.1		11.5	

^1^Means within a column for each developmental stage followed by the same letter are not significantly different (*P* > 0.05; All pairwise *t-*test)

^2^Observed number

^3^Developmental time (days)

^4^Maximum mean difference in developmental time among five host plants

**Table 2:** Survivorship (%; survived number/initial number) of *A. ipsilon* immatures according to host.

Host	Egg	Larva	Pupa (+prepupa) ^1^	Larva to adult
Napa cabbage	94.1 (744/791) a^2^	92.0 (92/100) a	92.4 (85/92) a	85.0 (85/100) a
Corn	93.3 (923/989) a	83.0 (83/100) a	73.5 (61/83) a	61.0 (61/100) b
Pepper	73.2 (572/781) b	58.0 (58/100) b	94.8 (55/58) a	55.0 (55/100) b
Bean	44.1 (164/372) c	35.0 (35/100) c	20.0 (7/35) b	7.0 (7/100) c
Perilla	93.1 (365/392) a	60.0 (60/100) ab	76.6 (46/60) a	46.0 (46/100) b

^1^In this analysis, prepupa stage was included in pupa stage

^2^Means within a column for each developmental stage followed by the same letter are not significantly different (*P* > 0.05; All pairwise chi-square test).

### 3.2 Effects of host on larvae and pupae

In the larva stage, the bean populations showed the longest time to complete its development and the lowest survivorship among the five hosts (Table 1, Table 2). In contrast, napa cabbage populations developed most rapidly to the prepupa stage with the highest survivorship (Table 1, Table 2). The prepupa stage of *A. ipsilon* was not significantly (*P* > 0.05) affected by hosts (Table 1, *F* = 2.12; df = 4, 102; *P* = 0.084). During the pupa stage, the developmental time of *A. ipsilon* was not consistent with its survivorship: the developmental rate was the lowest in corn, but survivorship was the lowest in bean. No statistically significant (*P* > 0.05) difference in survivorship of *A. ipsilon* was found between males and females during the pupa and prepupa stages: napa cabbage (*x*^2^ = 0.045; df = 1; *P* = 0.8318), corn (*x*^2^ = 0.024; df = 1; *P* = 0.8773), pepper (*x*^2^ = 0.085; df = 1; *P* = 0.7710), bean (*x*^2^ = 1.077; df = 1; *P* = 0.2994), and perilla (*x*^2^ = 0.420; df = 1; *P* = 0.5170). From eggs to adults, *A. ipsilon* populations reared on napa cabbage required the least time to complete their development with the highest survivorship (Table 1, Table 2). The differences in the developmental time of *A. ipsilon* by selected hosts were mainly attributed to the larval stage (Table 1). However, no statistical significance (*P* > 0.05) was found in the development (from eggs to adults) of the immatures of *A. ipsilon* between males and females: napa cabbage (*t* = −0.068; df = 83; *P* = 0.4730), corn (*t* = −0.898; df = 59; *P* = 0.1865), pepper (*t* = −1.244; df = 53; *P* = 0.1095), bean (*t* = 0.417; df = 5; *P* = 0.3470), and perilla (*t* = 0.785; df = 44; *P* = 0.2184).

Host species of *A. ipsilon* also affected the pupa weight. In both male and female, the pupa weight was the lowest in maize among all five hosts in this study (Table 3). Even though the average weight of *A. ipsilon* females was higher than males’ one regardless of hosts, statistically significant (*P* < 0.05) differences in pupa weight between males and females were determined by selected hosts: napa cabbage (*t* = −1.535; df = 98; P = 0.0640), corn (*t* = −0.873; df = 98; *P* = 0.1923), pepper (*t* = −3.729; df = 98; *P* = 0.0002), bean (*t* = −2.443; df = 98; *P* = 0.0082), and perilla (*t* = −2.183; df = 98; *P* = 0.0157).

**Table 3:** Pupa weights (g ± SE) of *A. ipsilon* according to hosts.

Sex	Napa cabbage	Corn	Pepper	Bean	Perilla
Male	0.40 ± 0.008 a	0.26 ± 0.010 b	0.39 ± 0.010 a	0.37 ± 0.008 a	0.37 ± 0.009 a
Female	0.42 ± 0.008 ab	0.28 ± 0.010 c	0.45 ± 0.013 a	0.40 ± 0.012 b	0.41 ± 0.013 b

^1^ Means within a row followed by the same letter are not significantly different (*P* > 0.05; *t*-test at 5% error rate).

### 3.3 Effects on host on adults

There was no statistically significant (*P* > 0.05) difference in longevity of *A. ipsilon* adults between males and females: napa cabbage (*t* = −0.987; df = 20; *P* = 0.1676), corn (*t* = −0.931; df = 20; *P* = 0.1814), pepper (*t* = 0.078; df = 28; *P* = 0.4692), bean (*t* = 0.574; df = 20; *P* = 0.2862), and perilla (*t* = −0.805; df = 20; *P* = 0.2153). However, host species affected the preoviposition and oviposition periods, and the oviposition number of female adults of *A. ipsilon* (Table 4). In napa cabbage, the female adults completed the preparation for oviposition most quickly and laid the most eggs for the longest time in this study (Table 4). The female adults in bean laid the fewest eggs during the shortest time among the five hosts (Table 4).

**Table 4:** Comparison of preoviposition (days ± SE) and oviposition periods (days ± SE), oviposition numbers (mean ± SE), and longevity (days ± SE) of *A. ipsilon* adults according to hosts.

Hosts	Preoviposition period	Oviposition period	Oviposition number	Longevity (male)	Longevity (female)
Napa cabbage	3.4 ± 0.40 b	7.7 ± 1.03 a	1656 ± 278.9 a	9.3 ± 0.56 b	10.4 ± 1.10 ab
Corn	5.0 ± 0.47 a	5.8 ± 1.04 ab	1064 ± 127.9 ab	13.3 ± 0.96 ab	14.5 ± 1.06 a
Pepper	4.7 ± 0.30 ab	3.7 ± 0.99 b	743 ± 141.1 bc	11.1 ± 1.18 ab	10.9 ± 1.24 ab
Bean	3.7 ± 0.24 ab	2.0 ± 0.60 b	242 ± 98.5 c	8.9 ± 0.99 b	8.1 ± 1.18 b
Perilla	4.6 ± .031 ab	8.9 ± 1.08 a	933 ± 132.2 bc	14.1 ± 1.38 a	18.7 ± 1.71 a

^1^ Means within a column followed by the same letter are not significantly different (*P* > 0.05; All pairwise *t-*test).

### 3.4 Relationships of host effects between biological factors

**Fig. 1:**
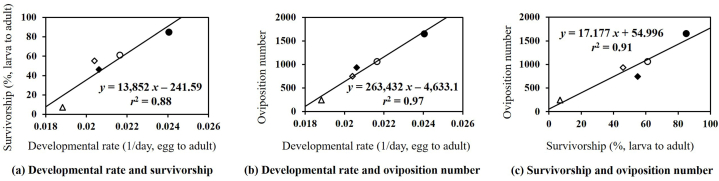
A few relationships of host effects between major biological factors of *A. ipsilon* (●: napa cabbage, ○: corn, ◆: perilla, ◇: pepper, △: bean): (a) developmental rate and survivorship of immatures; (b) developmental rate of immatures and oviposition number of female adults; (c) survivorship of immatures and oviposition number of female adults.

The host effect on each individual biological factor was positively corelated with one of the other biological factors (Fig. 1). This trend was predominantly observed between three major biological factors (i.e., developmental rate and survivorship of its immatures, and oviposition number of its female adults): developmental rate and survivorship (Fig. 1a), developmental rate and oviposition number (Fig. 1b), and survivorship and oviposition number (Fig. 1c). However, these trends were not clear at preoviposition period, oviposition period, and longevity.

## 4 Discussion

Host plants significantly (*P* < 0.05) affected the developmental time and survivorship of *A. ipsilon* immatures. Host effects were detected during all the developmental stages (i.e., egg, larva, and pupa) of *A. ipsilon*, even though the host effects were most pronounced at the larval stage. *A. ipsilon* populations completed its development from eggs to adults most quickly on napa cabbage with the highest survivorship among the five host plants studied, napa cabbage, corn, pepper, bean, and perilla. Host plants also affected reproductive factors. Significant (*P* < 0.05) host effects were found in preoviposition period, oviposition period and number, and longevity of *A. ipsilon* adults. Individuals growing on napa cabbages laid eggs more quickly than on other hosts. They also laid larger number of eggs over a longer duration and lived longer than on other hosts in this study. The corn population required more time to lay eggs than the other populations. However, the oviposited number of eggs on corns was statically (*P* < 0.05) same as the number on napa cabbage.

The egg stage of *A. ipsilon* showed significant (*P* < 0.05) host effects on both developmental time and survivorship in this study. Muştu et al. [12] reported that all the eggs of *A. ipsilon* hatched on forth days regardless of host plant (i.e., corn, potato, and sugar beet). This discrepancy might not be attributed to difference in host plants or experimental conditions between this study and their study [12]. The difference would be attributed to sample number and the level of adaptation to each host. This experiment used a minimum of 164 *A. ipsilon* eggs on beans and a maximum of 923 eggs on corns. Muştu et al. [12] used only 50 eggs on each host. In this study, almost all eggs hatched on forth days regardless of hosts as the results of Muştu et al. [12]. However, a few eggs hatched on fifth days. The number of hatched on fifth days were different according to host plants. This phenomenon made statistical differences (*P* < 0.05) in developmental time of *A. ipsilon* eggs. Muştu et al. [12] also adapted each population to each host during a single generation, which might not be an adequate adaptation period. The egg developmental rate could be affected by its previous host, lettuce. The complete development of *A. ipsilon* eggs did not require any food. The environmental conditions were similarly controlled in all host plants in this study. These conditions suggested that the developmental rate and survivorship of eggs might be determined by its mothers. Adaptation of female *A. ipsilon* adults on relatively better host plants and feeding adequately under favorable environmental conditions would lead to rapid hatching of eggs with higher survivorship compared with eggs of adapted females thriving on worse host plants in similar environmental conditions.

The larval development of *A. ipsilon* was heavily affected by host plants as reported by previous studies [8,9,11,12]. The differences in developmental time (days) of *A. ipsilon* immatures based on host plants were mainly determined during its larva stage even though this stage constitutes only half of the total developmental time. The phenomenon could be explained by the larval stage of *A. ipsilon*, which is the only active feeding period during its whole life. Its survivorship during the larval stage was affected by the host effects on the developmental time. Thus, *A. ipsilon* larvae developed faster and survived increasingly on specific host plants.

Interestingly, no statistically significant (*P* > 0.05) host effects of *A. ipsilon* prepupae were not found. However, the pupa including prepupa stage was affected by the host species. The pupa stage of *A. ipsilon* exhibited two distinct characteristics. First, the development of pupa did not mirror the host effects on the other developmental stages (i.e., egg and larva). The napa cabbage facilitated rapid completion of *A. ipsilon* eggs and larvae compared with the other five hosts in this study, which the development of pupae was delayed. Second, the developmental time of *A. ipsilon* by a selected host was not consistent with its survivorship on the host. The *A. ipsilon* pupae completed its development most quickly on corns, but showed moderate survivorship. By contrast, the pupae of pepper populations became adults at a moderate speed, although their survivorship was the highest among the five hosts. These findings indicate that the pupal development of *A. ipsilon* might not dependent on food quality alone. Corn plants contributed to the emergence of the smallest pupae among five hosts, with significant weight differences (*P* < 0.05) in both males and females compared to the other host plants. Thus, relatively low reproduction abilities of corn populations were expected, but their reproduction ability was the highest among the five host plants in this study.

The reproduction number of *A. ipsilon* females was strongly corelated with the periods of preoviposition and oviposition, but less with longevity. These phenomena were already reported by Muştu et al. [12]. This finding indicates that food quality would affect egg number during female lifetime. Under favorable environmental conditions, female adults would die after laying specific number of eggs. Except for longevity, reproductive factors including preoviposition period, oviposition period, and oviposition numbers of *A. ipsilon* were strongly corelated with the developmental rate and survivorship of its immatures according to hosts in this study. This phenomenon was already demonstrated in other Noctuidae moths [[14], [15], [16], [17]]. Thus, the host suitability of *A. ipsilon* could be determined based on the relative fitness of biological factors.

Currently, the management of *A. ipsilon* is heavily dependent on the use of systematic insecticides [18]. However, the side-effects of these insecticides were well known [[19], [20], [21]]. Thus, environmentally-friendly pesticides using entomopathogenic nematodes and plant extracts were developed to control *A. ipsilon* populations [18]. These insecticides are less effective compared with systemic insecticides for management of *A. ipsilon* [18]. An alternative management tactic was the use the trap plants for *A. ipsilon.* This study showed that napa cabbages and corns strongly facilitated the growth and survivorship of *A. ipsilon* populations. Even though the relationship between host suitability and host preference for *A. ipsilon* has yet to be demonstared, the positive relationship between the two factors is well known in whiteflies [22,23]. Because the host suitability of napa cabbage was substantially higher than that of other major horticulture crops in this study, it was worthwhile comparing the preferences of *A. ipsilon* for napa cabbages and other horticulture crops. Moreover, this study used only horticulture crops’ leaves even though the larvae of *A. ipsilon* could feed both their leaves and stems [3]. When the preference for napa cabbages is established with whole plants, its possible use as a trap plant for *A. ipsilon* is strongly increased.

## Author contribution statement

Young Su Lee: Conceived and designed the experiments; Contributed reagents, materials, analysis tools or data; Wrote the paper.

Hee-A Lee: Conceived and designed the experiments; Performed the experiments; Contributed reagents, materials, analysis tools or data.

Gil-Hah Kim: Conceived and designed the experiments; Contributed reagents, materials, analysis tools or data.

Sunghoon Baek: Conceived and designed the experiments; Analyzed and interpreted the data; Contributed reagents, materials, analysis tools or data; Wrote the paper.

## Data availability statement

Data will be made available on request.

## Additional information

No additional information is available for this paper.

## Declaration of competing interest

The authors declare that they have no known competing financial interests or personal relationships that could have appeared to influence the work reported in this paper.
